# A population-specific genomic reference panel for Taiwan: NHRI-RP-1

**DOI:** 10.1186/s12929-026-01261-y

**Published:** 2026-08-11

**Authors:** Kuang-Huan Cheng, Yi-Rong Chen, Ren-Hua Chung, Ming-Wei Lin, Hui-Ying Weng, Yuh-Ru Lin, Yung-Feng Lin, Jacob Shujui Hsu, Ralph Kirby, Shao-Yuan Chuang, Yu-Li Liu, Shiu-Feng Kathy Huang, Wei J. Chen, Chih-Cheng Hsu, Wayne Huey-Herng Sheu, Shih-Feng Tsai

**Affiliations:** 1 Institute of Bioinformatics and Structural Biology, National Tsing Hua University, Hsinchu, Taiwan; 2 Institute of Molecular and Genomic Medicine, National Health Research Institutes, Miaoli, Taiwan; 3 Institute of Population Health Sciences, National Health Research Institutes, Miaoli, Taiwan; 4 Institute of Public Health, National Yang Ming Chiao Tung University, Taipei, Taiwan; 5 Biomedical Industry Ph.D. Program, College of Life Sciences, National Yang Ming Chiao Tung University, Taipei, Taiwan; 6 Department of Medicine Research, Taipei Medical University Hospital, Taipei, Taiwan; 7 Graduate Institute of Medical Genomics and Proteomics, College of Medicine, National Taiwan University, Taipei, Taiwan; 8 Institute of Genetics, National Yang Ming Chiao Tung University, Taipei, Taiwan; 9 Center for Neuropsychiatric Research, National Health Research Institutes, Miaoli, Taiwan; 10 Pathology Core Laboratory, National Health Research Institutes, Miaoli, Taiwan; 11 Institute of Epidemiology and Preventive Medicine, College of Public Health, National Taiwan University, Taipei, Taiwan; 12 National Center for Geriatrics and Welfare, National Health Research Institutes, Yunlin, Taiwan; 13 Division of Endocrinology and Metabolism, Department of Internal Medicine, Taichung Veterans General Hospital, Taichung, Taiwan; 14 College of Pharmacy, Taipei Medical University, Taipei, Taiwan

**Keywords:** Reference panel, Imputation, Genome-wide association study

## Abstract

**Background:**

To enhance the efficiency of identifying rare variants within the Taiwanese population and to support genome-wide association studies (GWAS) and imputation studies for genetic risk prediction in the Han population, we have developed the National Health Research Institutes (NHRI) reference panel (NHRI-RP-1).

**Methods:**

NHRI-RP-1 is based on 2,561 whole genome sequences taken from the in-house NHRI datasets. Our objective was to optimize conditions of sample sizes (0.5K, 1K, 1.5K, 2K, 2.5K), minor allele frequency (MAF) thresholds (MAF ≥ 0.05, 0.01, 0.001, 2 × 10^–4^), and imputation quality (r^2^ ≥ 0, 0.3, 0.5) to build an aggregated genome reference panel for genetic medicine by comparing with worldwide references. Clinical applications and GWAS were then evaluated to demonstrate the capability of the reference panel.

**Results:**

Among different combinations of relevant parameters, the NHRI-RP-1 (with a 2,500-sample size, MAF ≥ 2 × 10^–4^, r^2^ ≥ 0) demonstrated a superior F1 score on local match, genotype concordance and r-squared, as compared to those using worldwide reference genomes, particularly for rare MAFs. Furthermore, NHRI-RP-1 achieved over 95% accuracy for nine pathogenic variants present in the Taiwan Biobank and 93.49% and 92.9% accuracy for imputing two *DRD1* variants.

**Conclusions:**

The use of different reference panels can influence the outcomes of GWAS. Our case studies demonstrate the utility of the NHRI-RP-1 for genetic medicine. Incorporating a population-specific panel such as NHRI-RP-1 can facilitate the development of prediction models using polygenic risk scores for diseases that are common in the Han population.

**Supplementary Information:**

The online version contains supplementary material available at 10.1186/s12929-026-01261-y.

## Background

Genotyping every single variant in the genome by whole-genome sequencing (WGS) is prohibitively expensive when millions of people are involved and at the moment is considered impractical for genetic medicine research. As a cost-effective alternative, researchers typically genotype only a subset of common variants using single-nucleotide polymorphism (SNP) arrays [1]. Imputation is then employed to predict ungenotyped variants by leveraging linkage disequilibrium (LD) patterns from large reference panels of phased haplotypes [2,3]. This strategy substantially increases genomic coverage at a fraction of the cost of WGS, while still providing sufficient resolution for GWAS that allow cross-cohort meta-analyses to be carried out, while supporting the fine-mapping of potential causal variants.

The quality of genotype imputation is influenced by several factors, including sample size, diversity, and variant density of the reference panel, design of genotyping array, and degree of genetic similarity between the reference and the study populations. In general, larger and denser reference panels that closely match the ancestry of the study population provide more accurate imputation, particularly for low-frequency and rare variants [1,4]. Widely used resources such as the 1000 Genomes (1KG) Project [5] and the Trans-Omics for Precision Medicine (TOPMed) reference panel [6] have substantially improved variant coverage and imputation quality. However, these panels are predominantly composed of individuals of non-Finnish European, Hispanic, or African ancestry, with comparatively limited representation of Asian populations. This underrepresentation reduces imputation accuracy in Asian cohorts and underscores the need for population-specific reference panels that will enable equitable genetic discovery across diverse populations [7,8].

In recent years, several efforts have focused on building population-specific WGS reference panels to improve imputation accuracy in underrepresented groups. For instance, the Northeast Asian Reference Database (NARD), constructed from 1,779 WGS samples across Korea, Mongolia, China, Japan, and Hong Kong, significantly improved imputation of variants with MAF < 1%, with r^2^ values increase from ~ 0.6 to ~ 0.8 compared to panels lacking regional representation [9]. The Japanese 1KG +  7 K panel, which combined the 1KG Project with 7,007 WGS samples from the Biobank Japan, increased imputation accuracy for low-frequency variants (MAF 0.5–5%) with mean r^2^ values improving from ~ 0.7 to ~ 0.9 compared to global panels [10]. Collectively, these studies demonstrate that incorporating population-matched WGS data into reference panels provides substantial improvements in imputation accuracy, particularly for low-frequency and rare variants, thereby increasing the resolution of association studies in specific populations.

In Taiwan, the Taiwan Biobank (TWB) reference panel, comprising 1,445 high-coverage whole-genome sequences, has been integrated into imputation pipelines and demonstrated superior imputation accuracy compared to the 1KG reference panel [11]. However, despite these advantages, the current TWB panel is limited by its sample size. A reference panel of 1,445 individuals is insufficient to fully capture the genetic diversity and low-frequency alleles present in the Taiwanese population. This limitation becomes particularly critical for imputing rare variants, where the accuracy strongly depends on the number of haplotypes represented within the panel. With a restricted sample size, many rare or population-specific variants may remain underrepresented or inaccurately imputed, thereby constraining downstream analyses such as GWAS and genetic risk prediction for common diseases.

The primary objective of the current study is to establish an effective strategy for enhancing imputation quality by expanding the sample size to 2,500 and by diversifying the MAF range. With the development of our new reference panel, we intend to compare the performance of TOPMed, 1KG 30X, 1KG phase 3 and TWB1.5K reference panels globally. Furthermore, this study aims to demonstrate the improvements in clinical applications in terms of the discovery of pathogenic rare variants and to elucidate how the use of different reference panels influences the outcomes of GWAS.

## Methods

### The datasets of the NHRI-RP-1

NHRI-RP-1 comprises 2,561 genomes of individuals experiencing healthy aging, disease, and cancer. The Healthy Aging Longitudinal Study in Taiwan (HALST) cohort recruited individuals aged 55 and above who were in good health. This cohort underwent home interviews and hospital-based clinical examinations every five years from 2009 [12]. Among the 5,664 participants in the HALST cohort, 1,022 completed WGS and provided electronic health records. Within the NHRI-RP-1, epilepsy, Fabry disease, obesity, hepatocellular carcinoma, and lung cancer patients were recruited, resulting in 1,539 WGS samples. The specimens of hepatocellular carcinoma and lung cancer were from the NHRI Biobank and the data of the community cohort was from TWB. All studied cohorts were approved by the respective Institutional Review Boards as indicated in the Ethics approval and consent to participate section.

### Genomesequencing and quality control

All datasets underwent sequencing at the NHRI Core Instrument Center utilizing the Illumina NovaSeq 6000 platform in Taiwan. WGS libraries were meticulously constructed employing the Illumina DNA PCR-Free Library Preparation Kit. Subsequently, variant calling was executed through the Nvidia Clara Parabricks v4.0.1 germline pipeline (https://docs.nvidia.com/clara/parabricks/4.0.1/index.html) [13]. Subsequently, the aligned datasets underwent quality control by the GATK Variant Quality Score Recalibration (VQSR) pipeline to effectively eliminate probable artifacts (https://gatk.broadinstitute.org/hc/en-us/articles/360035531612-Variant-Quality-Score-Recalibration-VQSR) [14].

### NHRI-RP-1 construction

Our NHRI-RP-1 pipeline was constructed using the GATK Best Practices Workflow (GATK v4.3), which comprises two core components: (1) pre-processing, which transforms raw reads into analysis-ready mapped reads, and (2) variant discovery, which processes analysis-ready reads to identify variants (https://gatk.broadinstitute.org/hc/en-us) [15]. The pre-processing and variant calling segment of the pipeline, from BWA to GATK Haplotype Caller, was implemented as the Single-Sample Calling pipeline. The individual genomic variant call format (gVCF) files were aggregated into multisample gVCF files by creating GenomicsDB and exporting a merged gVCF file. The workflow steps from Genotype GVCFs, VariantFiltration, VariantRecalibrator, and ApplyRecalibration, which operate on datasets, were implemented as the Joint Analysis pipeline.

To develop our candidate NHRI-RP-1, we randomly sampled individuals to generate five reference-panel sizes (0.5K, 1 K, 1.5K, 2 K, and 2.5K). Subsequently, we constructed chromosome-specific VCFs and performed quality-control procedures independently. Utilizing bcftools filter, we excluded SNPs with missing genotypes (F_MISSING > 0.05) and stratified variants by MAF into four thresholds (MAF ≥ 0.05, 0.01, 0.001, and 2 × 10^–4^) [16]. We restricted the dataset to bi-allelic SNPs and excluding indels according to the software guidelines [16].

### Imputation testing of the CVDFACTS study samples

In our imputation testing, we utilized the Cardiovascular Disease Risk Factor Two-Township Study in Taiwan (CVDFACTS), which encompassed subjects from two cohorts situated in northern and southern Taiwan [17]. This study enrolled 2,877 subjects aged 18 and above whose blood samples were subjected to analysis for various factors. We selected 55 CVDFACTS Study samples from the TWB 2.0 array and incorporated WGS data into our analysis. All array data was partitioned by chromosomes under the control of the imputation servers as follows:

The TOPMed Imputation Reference Panel (r^2^ version) comprised a heterogeneous reference sample of 97,256 individuals and 308,107,085 genetic variants distributed across the 22 autosomes and the X chromosome. This dataset was processed using Eagle v2.4 for phasing and Minimac 4 for imputation through the TOPMed imputation server, employing a r-squared filter of 0.3 [6,18].

The Michigan imputation server accommodated a diverse range of reference panels, encompassing genotype and HLA imputation. In this study, we selected 1KG Phase 3 (Version 5) and 1KG Phase 3 30 × as reference panels [5]. We processed them using Eagle v2.4 for phasing and Minimac 4 for imputation, employing a r-squared filter of 0.3 [18].

The TWB imputation server (https://twbimpute.biobank.org.tw/index.html#!pages/home) provided the Taiwan Biobank 1.5k reference panel (TWB1.5K hereafter) for for genotype imputation service using Eagle v2.4 for phasing and Minimac 4 for imputation, employing a r-squared filter of 0.3 [11].

All array datasets were phased and imputed using Beagle v5.4 by our designed NHRI-RP-1 with sample sizes of 0.5K, 1 K, 1.5K, 2 K, and 2.5K. Each NHRI-RP-1 had four MAF thresholds (MAF ≥ 0.05, 0.01, 0.001, and 2 × 10^–4^). After imputation, SNPs from the imputed data had already predicted an r-squared score for quality. We set the r-squared score below 0, 0.3, and 0.5 for each criterion of the imputed data. The completed imputed individual data with diverse criteria of NHRI-RP-1 were then compared with the WGS data for performance evaluation.

### Imputation performance analysis

Our matching strategies used ‘local match’ and ‘genotype match’ comparing the imputed and WGS data [19]. For local match, we compared the performance metrics, including true positive (TP), false positive (FP), and false negative (FN) to assess the accuracy of variant site even if alleles or genotypes differ [19]. Sensitivity measures the ability to detect known variant site or the absence of FNs. Precision, similarly, measures the ability to correctly identify the absence of variant site or FPs. The F1 score is the harmonic mean of precision and sensitivity. Mathematically, it is given by:

$${\mathrm{Sensitivity}}\, = \,{{{\mathrm{True}}\,{\mathrm{Positive}}\,\left( {{\mathrm{TP}}} \right)} \mathord{\left/ {\vphantom {{{\mathrm{True}}\,{\mathrm{Positive}}\,\left( {{\mathrm{TP}}} \right)} {\left( {{\mathrm{True}}\,{\mathrm{Positive}}\,\left( {{\mathrm{TP}}} \right)\, + \,{\mathrm{False}}\,{\mathrm{Negative}}\,\left( {{\mathrm{FN}}} \right)} \right)}}} \right. \kern-0pt} {\left( {{\mathrm{True}}\,{\mathrm{Positive}}\,\left( {{\mathrm{TP}}} \right)\, + \,{\mathrm{False}}\,{\mathrm{Negative}}\,\left( {{\mathrm{FN}}} \right)} \right)}}$$

$${\mathrm{Precision}}\, = \,{{{\mathrm{True}}\,{\mathrm{Positive}}\,\left( {{\mathrm{TP}}} \right)} \mathord{\left/ {\vphantom {{{\mathrm{True}}\,{\mathrm{Positive}}\,\left( {{\mathrm{TP}}} \right)} {\left( {{\mathrm{True}}\,{\mathrm{Positive}}\,\left( {{\mathrm{TP}}} \right)\, + \,{\mathrm{False}}\,{\mathrm{Positive}}\,\left( {{\mathrm{FP}}} \right)} \right)}}} \right. \kern-0pt} {\left( {{\mathrm{True}}\,{\mathrm{Positive}}\,\left( {{\mathrm{TP}}} \right)\, + \,{\mathrm{False}}\,{\mathrm{Positive}}\,\left( {{\mathrm{FP}}} \right)} \right)}}$$

$${\mathrm{F1}}\,{\mathrm{score}}\, = \,{2}\, \times \,{{\left( {{\mathrm{Precision}}\, \times \,{\mathrm{Sensitivity}}} \right)} \mathord{\left/ {\vphantom {{\left( {{\mathrm{Precision}}\, \times \,{\mathrm{Sensitivity}}} \right)} {\left( {{\mathrm{Precision}}\, + \,{\mathrm{Sensitivity}}} \right)}}} \right. \kern-0pt} {\left( {{\mathrm{Precision}}\, + \,{\mathrm{Sensitivity}}} \right)}}$$

For genotype match which sites with matching alleles and genotypes are counted as TPs, we assessed both genotype concordance and the empirical dosage r^2^, defined as the squared Pearson correlation between imputed alternate allele dosages and WGS validated genotypes [19]. Analyses were performed using the vcfR package in R. WGS genotypes were treated as the gold standard and encoded as discrete numerical values: 0 for homozygous reference (0/0), 1 for heterozygous (0/1 or 1/0), and 2 for homozygous alternate (1/1). Imputed genotypes were evaluated using the expected alternate allele dosage (DS field), which ranges continuously from 0.0 to 2.0 and represents the expected number of alternate alleles. Genotype concordance was defined as the proportion of variants where the absolute difference between the discrete WGS genotype and the imputed dosage was less than 0.5. The global empirical r^2^ was calculated by squaring the Pearson correlation coefficient between WGS genotypes and imputed dosages across all variants.

To further characterize imputation performance, empirical r^2^ values were summarized by chromosome and by MAF intervals (7 intervals: (0.01%, 1%], (1%, 5%], (5%, 10%], (10%, 20%], (20%, 30%], (30%, 40%], (40%, 50%]). Chromosome-specific performance was calculated by aggregating variants within each autosome and computing the mean r^2^ across variants. To evaluate the impact of allele frequency on imputation accuracy, variants were stratified into predefined MAF intervals based on WGS allele frequencies, and the mean r^2^ and standard error were calculated within each bin. Because reference panels differ in variant coverage, we additionally calculated a coverage-weighted global r^2^ by summing the r^2^ values of common variants and dividing by the total number of variants detected in the WGS dataset, thereby penalizing variants absent from the reference panels.

To select our candidate, NHRI-RP-1, we imputed our 55 CVDFACTS Study TWB2.0 array data using various criteria of NHRI-RP-1 and compared them with the WGS data. Subsequently, we identified the candidates and compared the performance with TOPMed, 1KG 30X, and 1KG phase 3 reference panels.

### Clinical applications

Two studies showcased the utility of NHRI-RP-1. First, a total of 1,395 samples genotyped using the TWB 2.0 array were integrated with WGS data [20]. Genotype imputation was conducted with the NHRI-RP-1 pipeline, and the imputed pathogenic and likely pathogenic (P/LP) rare variants were compared with the corresponding WGS-derived genotypes for validation. These variants were associated with hereditary breast and ovarian cancer, Lynch syndrome, familial hypercholesterolemia, hereditary hearing loss, and cerebral autosomal dominant arteriopathy with subcortical infarcts and leukoencephalopathy (CADASIL). Subsequently, we evaluated performance using both number of FPs and FNs and genotype concordance. In the second case, we collaborated with Dr. Yu-Li Liu from the NHRI to predict two *DRD1* variants from the 338 TWB 2.0 array samples [21]. These samples were imputed utilizing the 1KG phase 3 and NHRI-RP-1 pipelines, and the resulting genotypes were compared to the Taqman sequence results for the assessment of predicted accuracy.

### Genome-wide association study

We performed a GWAS using the CVDFACTS Study longitudinal cohort with a hypertension phenotype, comprising 788 cases and 531 controls. Genotyping was conducted with the TWB 2.0 array, and samples were imputed separately using the 1KG Phase 3 reference panel and the NHRI-RP-1 pipeline. Quality control procedures included excluding individuals with a genotype missing rate > 0.02 or heterozygosity deviating by more than ± 3 standard deviations from the mean. To account for relatedness, individuals with pairwise identity-by-descent > 0.25 were excluded using PLINK [22]. SNP-level quality control excluded variants with Hardy–Weinberg equilibrium p < 1 × 10^–6^ or MAF < 0.01. Association testing was performed using logistic regression. Visualization of GWAS results was conducted with the R package CMplot, and Manhattan plots were generated with genome-wide significance and suggestive thresholds set at 5 × 10^–8^ and 1 × 10^–5^, respectively.

To assess the potential impact of population stratification and systematic bias on the GWAS results, we calculated the genomic inflation factor (λ) for each reference panel. The genomic inflation factor was computed by dividing the median of the observed chi-squared association test statistics by the theoretical median of a chi-squared distribution with one degree of freedom, which represents the expected value under the null hypothesis. When GWAS results were reported as p-values, they were first converted to chi-squared statistics, and the median chi-squared value was subsequently calculated [23].

## Results

### Establishing a Taiwanese genomic reference panel

**Fig. 1:**
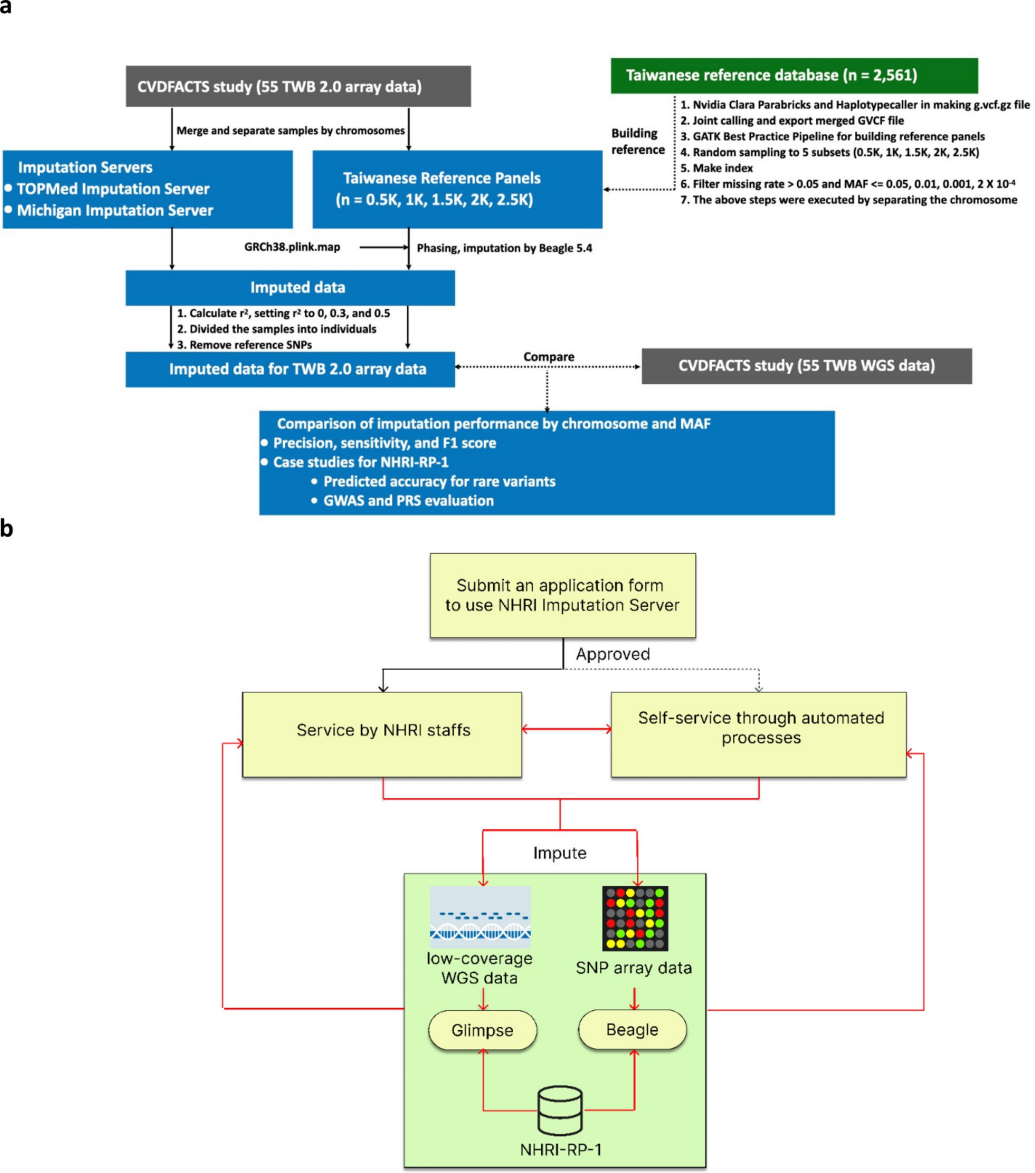
**a** Workflow for genotype imputation evaluation. This diagram details the construction and evaluation of custom Taiwanese reference panels for genotype imputation using Taiwan Biobank (TWB) 2.0 array data. A large Taiwanese WGS reference database (n = 2,561) is processed through variant calling and filtering, generating five subsampled reference panels (0.5–2.5K samples). A separate dataset of TWB 2.0 array from 55 samples of the CVDFACTS Study is then imputed in two ways: via public imputation servers (TOPMed and Michigan) and against the locally built Taiwanese panels using Beagle 5.4. The imputed data is compared against the WGS data from 55 samples of the CVDFACTS Study (acting as the true genotypes) to assess performance. The evaluation focuses on precision, sensitivity, and F1 score across different chromosomes and minor allele frequencies (MAFs). The validation includes case studies on rare variant accuracy and the impact on GWAS results. **b** Access to the NHRI Imputation Server. To make the NHRI-RP-1 available to the academic users, a website (http://imgm.healthhub.tw/NHRI-RP-1.html) was created so that the potential users can submit their requests. Currently, we are providing the imputation service by the NHRI staffs. A self-service mechanism is being established through the website to automate the imputation service. The NHRI Imputation Server will handle inputs in the form of low-coverage WGS as well as SNP array data

The overall objective and approach for developing NHRI-RP-1 are shown in Fig. 1. We have adhered to the established workflow (Fig. 1a) and randomly partitioned the available reference samples (n = 2,561) into five subsets of progressively increasing size (0.5K, 1 K, 1.5K, 2 K, and 2.5K) to systematically assess imputation performance relative to the WGS data. To promote the use of NHRI-RP-1 for genetic medicine research, we have created a website interface (http://imgm.healthhub.tw/NHRI-RP-1.html) for researchers to submit their requests for imputation service (Fig. 1b).

### Sample size and reference panel performance

**Fig. 2:**
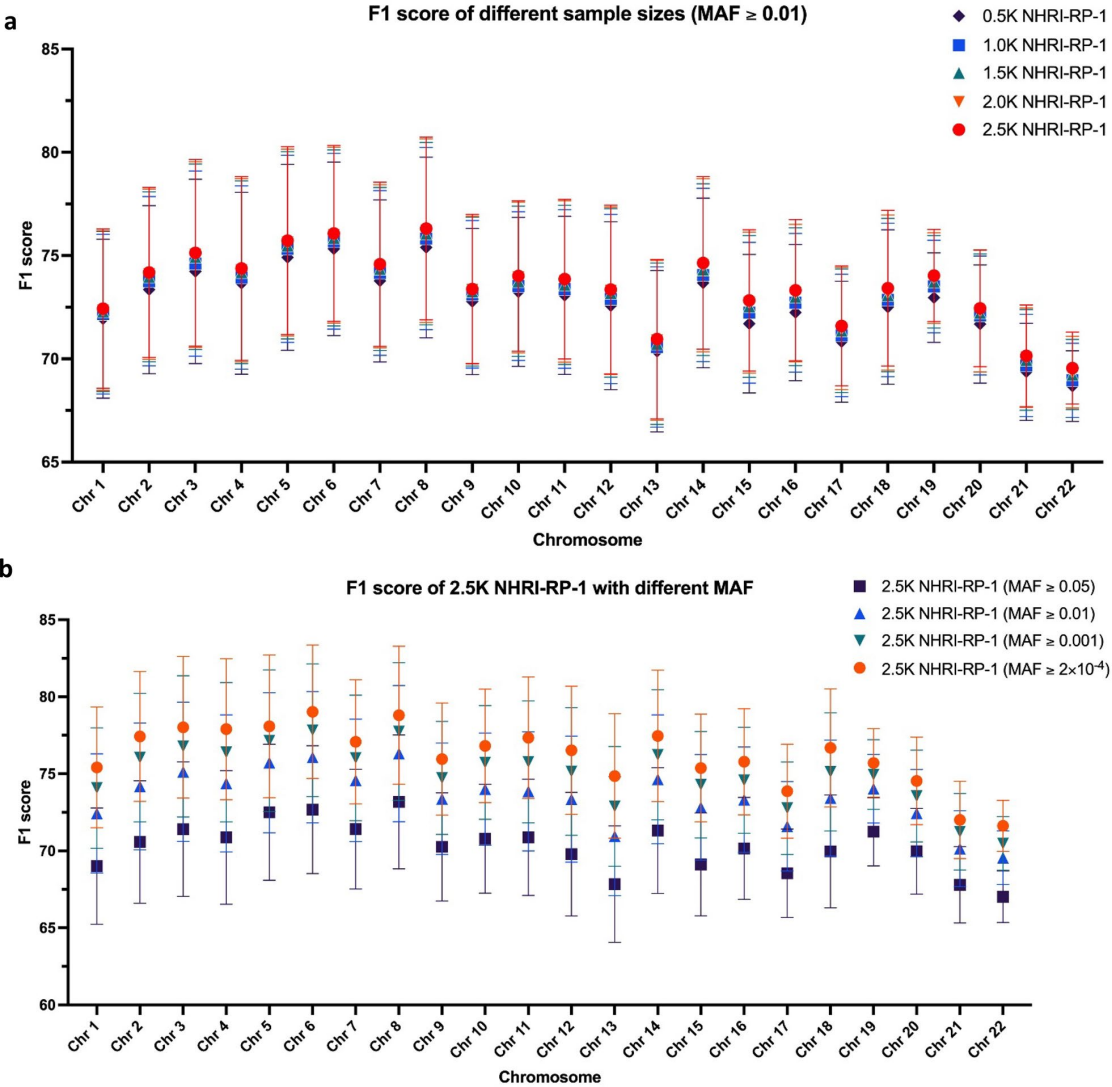
F1 score comparison using NHRI-RP-1 across different sample sizes and MAFs. F1 scores were generated across chromosomes with the NHRI-RP-1 dataset under various conditions. **a** sample sizes (0.5K, 1 K, 1.5K, 2 K, and 2.5K) and **b** variants with minor allele frequencies below thresholds (0.05, 0.01, 0.001, and 2 × 10^–4^)

Figure 2a demonstrates that increasing the sample size enhances precision, sensitivity, and F1 score, while at the same time minimizing variability (Fig. 2a, Additional file 1). The F1 scores generally ranged between 70 and 80, although performance varied from chromosome to chromosome. Larger chromosomes, such as chromosomes 8 and 19, exhibited relatively higher scores, while smaller chromosomes, like chromosomes 21 and 22, yielded lower scores across all sample sizes. Together, these findings suggest that larger sample sizes provide more stable and reliable results for variant detection, while the chromosome-specific differences indicate inherent variability in detection performance across the genome.

### MAF stratification and accuracy

Subsequently, for the 2.5K NHRI-RP-1 panel, we further stratified variants by MAF into four thresholds (MAF ≥ 0.05, 0.01, 0.001, and 2 × 10^–4^). The analysis revealed that the 2.5K NHRI-RP-1 at a MAF ≥ of 2 × 10^–4^ achieved the highest F1 scores and sensitivity across all chromosomes, followed by MAF ≥ 0.001. In contrast, variants with MAF ≥ 0.05 consistently demonstrated the lowest F1 scores (Fig. 2b, Additional file 2b). Precision values were generally high, exceeding 90% across all MAF thresholds; however, the overall trend of improved performance with lower MAF thresholds remained evident (Additional file 2a). These findings suggest that applying a more stringent, lower MAF threshold (e.g., ≥ 2 × 10^–4^) enhances both the accuracy and comprehensiveness of imputation, largely due to improved detection of rare variants.

### Imputation quality assessment and r^2^ thresholds

**Fig. 3:**
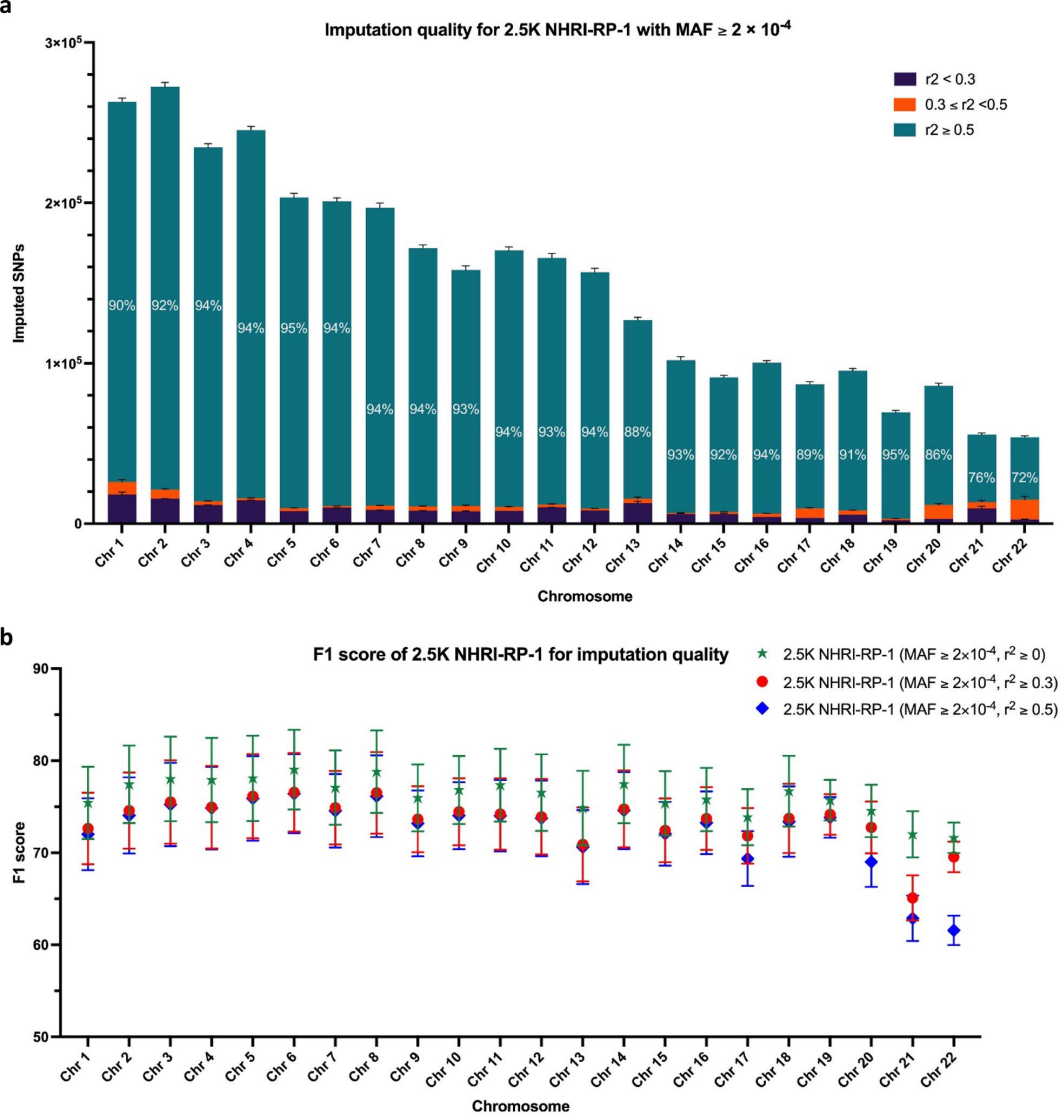
Imputation quality and F1 scores comparison after imputing 2.5K NHRI-RP-1 with a MAF below 2 × 10^–4^. **a** The imputation quality is evaluated using different r-squared values for SNPs on each chromosome. Purple: r-squared values less than 0.3; orange: r-squared values between 0.3 and 0.5; green: r-squared values greater than 0.5. **b** A comparative analysis of F1 scores with the 2.5K NHRI-RP-1 dataset is conducted, focusing on variants characterized by a MAF of below 2 × 10^–4^. This analysis is performed using different criteria of r-squared values for SNPs. Green: r-squared values greater than 0; red: r-squared values greater than 0.3; blue: r-squared values greater than 0.5

After fixing the basic parameters of sample size and MAF, we next evaluated the imputation quality of the putative NHRI-RP-1 dataset, taking into account the threshold to report a variant. As shown in Fig. 3a, at r^2^ ≥ 0.5, the majority of imputed SNPs achieved high accuracy, ranging from 95% on chromosome 5–72% on chromosome 22. Lower performance was observed on smaller chromosomes such as 21 and 22. The F1 score panel shows consistent imputation performance across chromosomes, with values ranging from 70 to 80, though they were slightly lower on chromosomes 21 and 22. At r^2^ ≥ 0, all imputed variants were included, resulting in higher sensitivity and F1 scores but with some low-confidence SNPs retained. As the threshold increased to r^2^ ≥ 0.3 and r^2^ ≥ 0.5, the number of retained SNPs decreased, leading to lower F1 scores, especially on smaller chromosomes (Fig. 3b).

Overall, imputation quality was robust and consistent, but with reduced performance on smaller chromosomes. Moving from r^2^ ≥ 0.5 to r^2^ ≥ 0 reflects a trade-off of precision for sensitivity. Precision remained high (> 90%) across all thresholds (Additional file 3a). Whereas stringent criterion (r^2^ ≥ 0.5) ensures greater confidence but reduces sensitivity, r^2^ ≥ 0 offers broader variant inclusion and higher sensitivity (Additional file 3b). For our optimized reference panel, we chose r^2^ ≥ 0 to improve coverage of rare variants, making it more suitable for discovery phase rather than for confirmatory analysis.

### Performance of the NHRI-RP-1 panel in comparison to other reference panels

#### Local match comparison and MAF influence

**Fig. 4:**
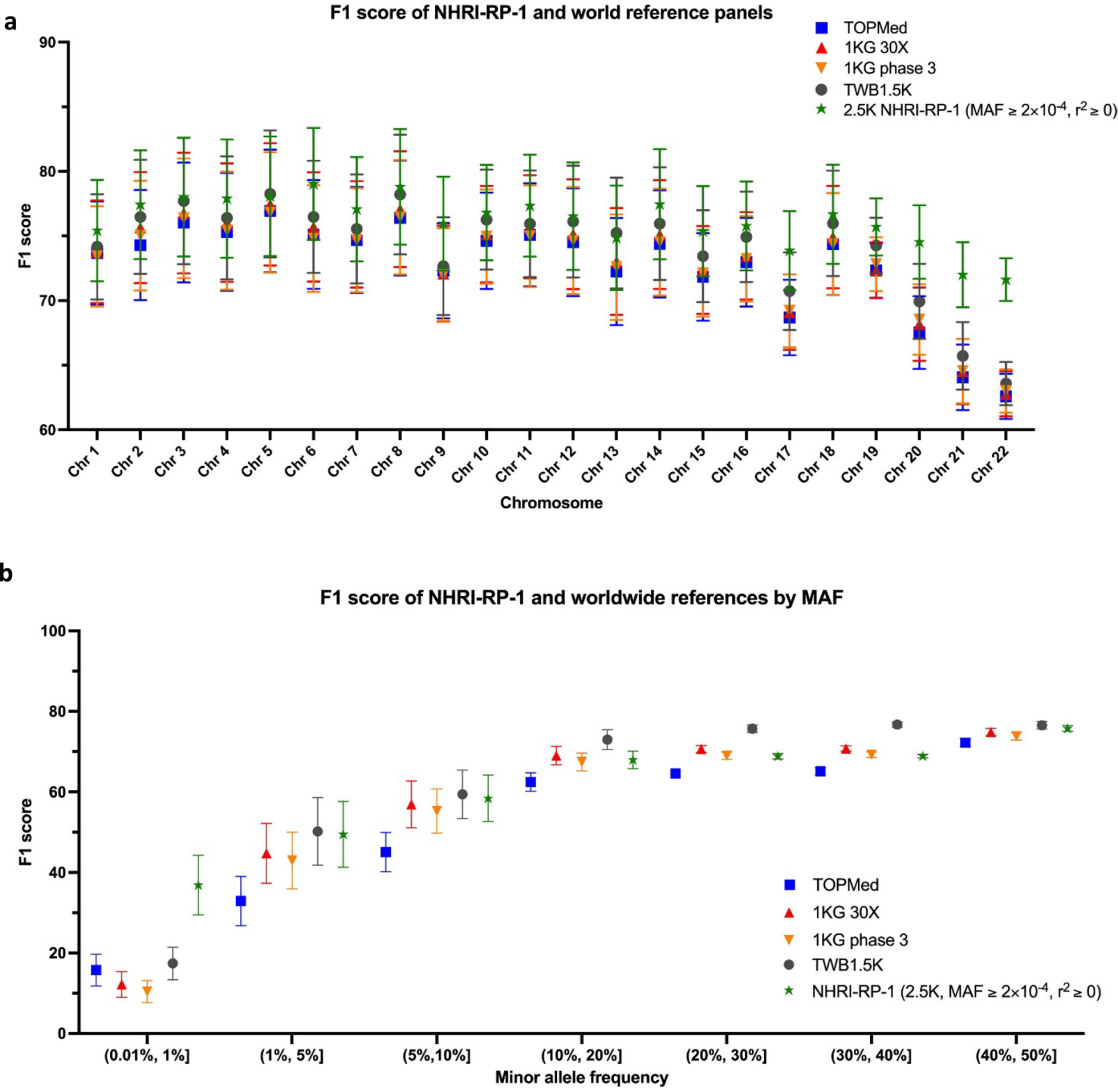
F1 score comparison of NHRI-RP-1, TOPMed, 1KG, and TWB1.5K reference panels. Local match was applied to evaluate the imputation performance. **a** by chromosomes and **b** by MAF intervals

For the local match comparison, our reference panel, NHRI-RP-1, demonstrated superior performance in precision, sensitivity, and F1 score, compared to global reference panels (TOPMed, 1KG 30X, 1KG Phase 3, and TWB1.5K) across most chromosomes (Fig. 4a, Additional file 4). However, performance declined markedly for chromosomes 19 through 22, where F1 scores dropped to 60–70. Performance metrics were also strongly influenced by MAF. As variants transited from rare to common (i.e., across increasing MAF intervals), precision, sensitivity, and F1 scores improved substantially for all panels, including NHRI-RP-1 (Fig. 4b, Additional file 5). For very rare variants (MAF < 1%), performance was consistently low across all panels, with F1 scores falling below 20 for MAF of 0.01–1%. By contrast, as MAF rose to 40–50%, F1 scores exceeded 60 for all panels. These findings underscore the challenges of accurately imputing very rare variants, irrespective of the reference panels employed.

#### Genotype match comparison

**Fig. 5:**
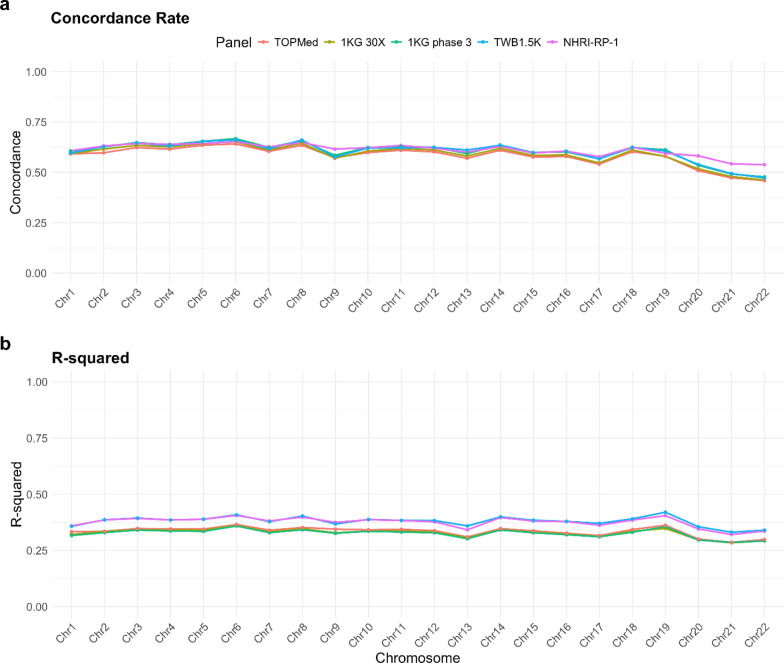
Genotype imputation performance across autosomes. Local reference panels (TWB1.5K and NHRI-RP-1) were compared against the global reference panels (TOPMed, 1KG 30X, and 1KG phase 3) using two metrics: **a** Genotype concordance rate, **b** Empirical dosage r-squared

For the genotype match comparison, Fig. 5a illustrates that the NHRI-RP-1 maintained a stable genotype concordance rate, generally ranging between 0.55 and 0.65 across the genome. While its performance was comparable to other reference panels across most chromosomes, it demonstrated a distinct advantage on the smaller chromosomes—specifically chromosomes 20, 21, and 22—where it avoided the sharp performance decline seen in the global reference panels (TOPMed and 1KG). Consistently, Fig. 5b shows that NHRI-RP-1 (alongside the TWB1.5K) achieved significantly higher empirical r^2^ values than the global reference panels across all chromosomes.

**Fig. 6:**
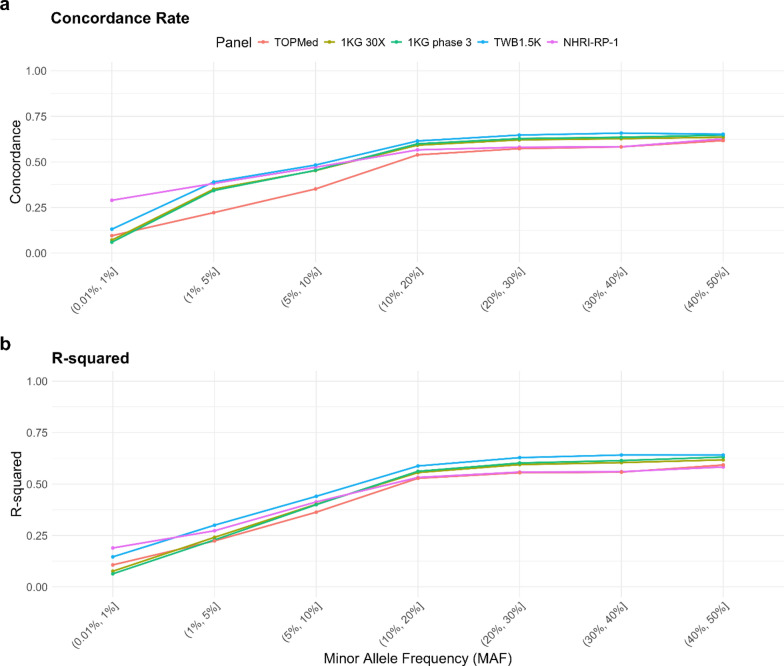
Genotype imputation performance stratified by seven MAF intervals. Local reference panels (TWB1.5K and NHRI-RP-1) were compared against the global reference panels (TOPMed, 1KG 30X, and 1KG phase 3) using two metrics: **a** Genotype concordance rate, **b** Empirical dosage r-squared

When imputation performance was stratified by MAF, a clear positive relationship between allele frequency and imputation accuracy was observed across all reference panels. However, a pronounced performance gap emerged in the rare-variant range (MAF < 1%). In this spectrum, NHRI-RP-1 demonstrated a substantial advantage, achieving a concordance rate of nearly 0.3 (Fig. 6a) and an r^2^ value of approximately 0.2 (Fig. 6b). In contrast, the global reference panels (1KG and TOPMed) showed a marked decline, with r^2^ values falling below 0.1.

Taking together, these findings emphasize the importance of population-specific reference panels for accurate imputation of rare variants, which are often critical in studies of complex disease architecture and precision medicine.

#### Applications of the reference panel for imputing SNP array data

To evaluate the utility of imputation algorithms in clinical applications, we conducted two studies. In the first study, we obtained 1,395 samples from the TWB 2.0 array and integrated the associated WGS data into our analysis. Our primary objective was to predict the imputation accuracy of seven P/LP rare variants with a MAF exceeding 0.1% within the Taiwanese population (data from a related study accepted for publication at Genetics in Medicine Open). These samples were imputed using the NHRI-RP-1 imputation pipeline, and the resulting genotypes were compared to the WGS data.

To account for the high proportion of non-carriers in rare variant analysis, we evaluated performance using both numbers of FPs and FNs and genotype concordance. The NHRI-RP-1 demonstrates exceptionally high imputation accuracy for clinically significant P/LP variants, with genotype-level concordance ranging from 96.85 to 100% (Table 1). For genes such as *APOB*, and *NOTCH3*, which are associated with familial hypercholesterolemia, and CADASIL, respectively, the imputation accuracy reaches a perfect 100% with zero FPs or FNs. While the accuracy remained high for the *LDLR* and *GJB2* variants (96.85%–99.86%), minor discrepancies were noted; for instance, the *GJB2* c.109G > A variant showed 34 FPs and 10 FNs, resulting in the lowest observed concordance of 96.85%. However, the two frameshift variants in *GJB2* (c.235del and c.299_300del) showed no FPs (Table 1). These findings indicate that the NHRI-RP-1 is a highly reliable tool for identifying disease-associated variants, even at very low frequencies, within a population-specific context.

**Table 1:** Assessed imputation accuracy of rare P/LP variants in key genes using TWB data

Gene	HGVS descriptions	Function	MAF*	Carriers in TWB (WGS)	Number of correctly imputed	Number of false positives	Number of false negatives	Genotype-level concordance (%)
*APOB*	NM_000384.3(APOB):c.10579C > T (p.Arg3527Trp)	Missense variant	0.001	9	1395	0	0	100
*LDLR*	NM_000527.5(LDLR):c.268G > A (p.Asp90Asn)	Missense variant	0.001	5	1393	1	1	99.86
*LDLR*	NM_000527.5(LDLR):c.1747C > T (p.His583Tyr)	Missense variant	0.002	6	1393	2	0	99.86
*NOTCH3*	NM_000435.3(NOTCH3):c.1630C > T (p.Arg544Cys)	Missense variant	0.004	11	1395	0	0	100
*GJB2*	NM_004004.6(GJB2):c.109G > A (p.Val37Ile)	Missense variant	0.09	234	1351	34	10	96.85
*GJB2*	NM_004004.6(GJB2):c.235del (p.Leu79fs)	Frameshift variant	0.005	17	1378	0	17	98.78
*GJB2*	NM_004004.6(GJB2):c.299_300del (p.His100fs)	Frameshift variant	0.001	5	1390	0	5	99.64

*HGVS* Human Genome Variation Society, *P/LP* Pathogenic or likely pathogenic, *TWB* Taiwan Biobank, *MAF* Minor allele frequency

^*^The MAF results were conducted in our in-house NHRI cohort (n = 2,473)

In the second investigation, we employed a 338 TWB 2.0 SNP array dataset from Dr. Yu-Li Liu’s laboratory to predict the accuracy of two *DRD1* variants [21]. The NHRI-RP-1demonstrated superior imputation accuracy for the *DRD1* gene variants compared to the 1KG phase 3 reference panel in the Taiwanese population (Table 2). For the variant at position 175440166 (rs12518222), the NHRI-RP-1achieved an accuracy of 93.49%, surpassing the 1KG phase 3 reference panel’s 90.53%. Similarly, for the variant at position 175440896 (rs4867798), the accuracy of NHRI-RP-1 was 92.90%, again surpassing the 1KG phase 3 reference panel’s 91.42% (Table 2). This suggests that the population-specific NHRI-RP-1 is more effective when imputing genetic variants within the Taiwanese population, likely due to its superior representation of local genetic diversity and unique haplotype structures.

**Table 2:** Compared *DRD1* imputation accuracy in the Taiwanese cohort using 1KG Phase 3 versus NHRI-RP-1 datasets

Gene	Position in GRCh38	rsID	cDNA	TaqMan Seq (Ref/Alt)	Imputation predicted accuracy (%)	
					1KG p3 (%)	2.5K NHRI-RP-1 (%)
*DRD1*	175440166	rs12518222	c.*1593G > A	C/T	90.53	93.49
*DRD1*	175440896	rs4867798	c.*863A > G	T/C	91.42	92.90

#### Impact of reference panel choice on GWAS outcome

**Fig. 7:**
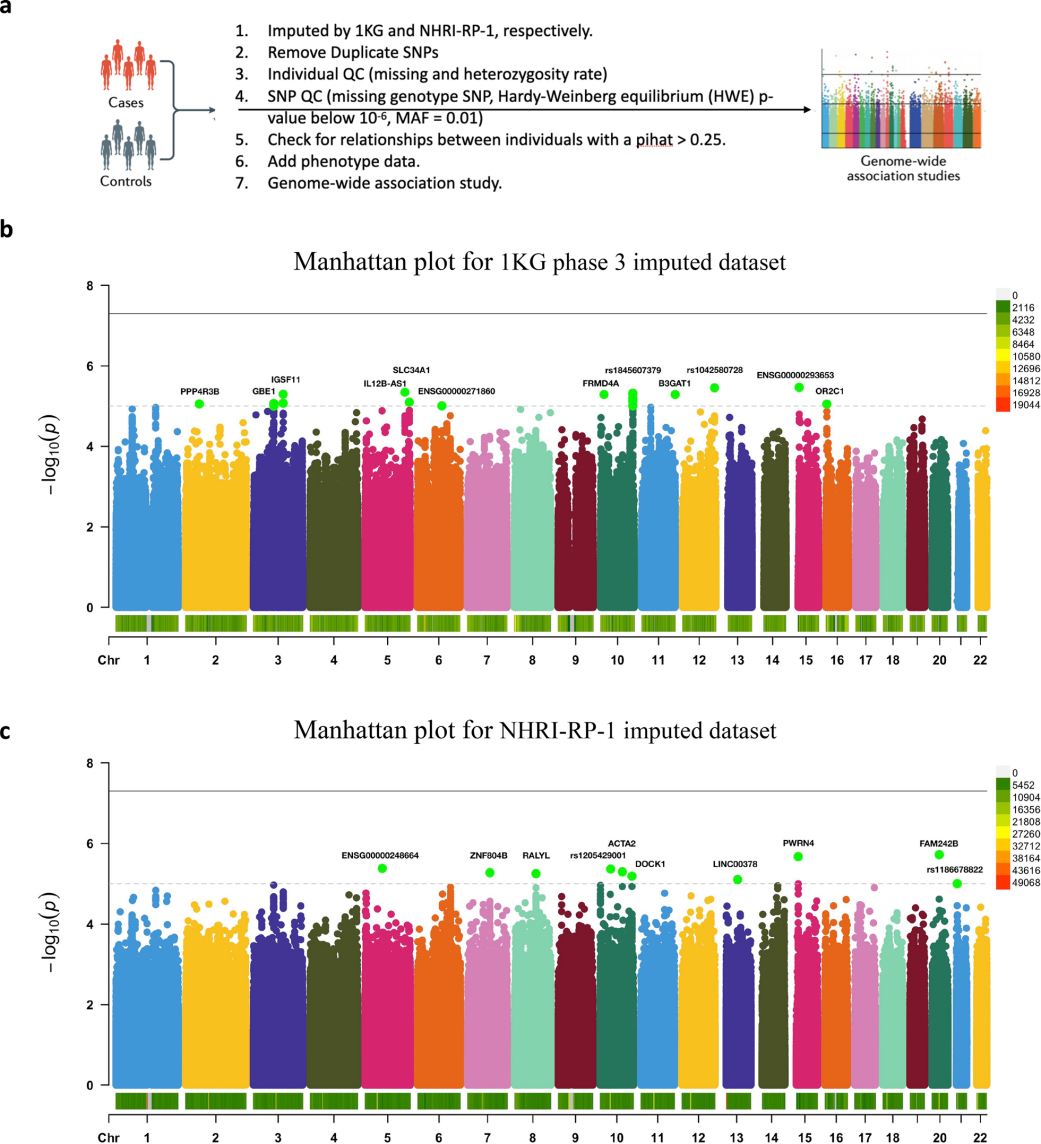
Workflow and analyses of CVDFACTS cohorts for genome-wide association studies (GWAS). **a** The cohort comprises 788 hypertension cases and 531 controls, each with TWB array data imputed from the 1KG phase 3 and NHRI-RP-1 reference panels, respectively. All imputed data undergo rigorous quality control processes. **b** Manhattan plot for the GWAS results by 1KG phase 3 reference panel. **c** Manhattan plot for the GWAS results by NHRI-RP-1

Finally, to demonstrate the application of the optimized reference model for real-world genetic research, a GWAS was conducted using the CVDFACTS Study longitudinal cohort, comprising 788 cases and 531 controls. Genotyping was performed with the TWB 2.0 array, and imputation was carried out using both the 1KG Phase 3 reference panel and the NHRI-RP-1 pipeline to generate the variant dataset for quality control and downstream analyses (Fig. 7a). The Manhattan plot based on the 1KG Phase 3 reference panel (Fig. 7b) revealed a substantial number of variants exceeding the genome-wide significance threshold (− log10(P) = 5), particularly on chromosomes 2, 3, 5, 10, 11, 12, 15, and 16. In contrast, the plot based on the NHRI-RP-1 (Fig. 7c) showed a markedly reduced number of significant associations, with only limited variants on chromosomes 5, 7, 8, 10, 13, 15, 20 and 21 surpassing or approaching the threshold.

To further evaluate the statistical integrity of these findings, we calculated the genomic inflation factor (λ) for both sets of results. The 1KG Phase 3 reference panel yielded a λ of 0.8762, while the NHRI-RP-1 produced a more conservative λ of 0.754. These values indicate a degree of genomic deflation rather than inflation, suggesting that the association models are highly conservative and well-controlled against false-positive signals arising from population stratification. The more pronounced deflation observed with the NHRI-RP-1 aligns with its superior population specificity for the Taiwanese cohort, effectively filtering out non-specific haplotypes that might otherwise contribute to statistical noise observed in broader, multi-ethnic reference panels. While the 1KG Phase 3 reference panel identified a greater number of variants exceeding the significance threshold, the lower λ and focused associations of the NHRI-RP-1 pipeline suggest that it provides a more stringent representation of the studied cohort's unique genetic architecture. These results highlight how the choice of reference panel can substantially influence a GWAS outcome and underscores the importance of considering multiple reference panels when developing polygenic risk prediction models.

## Discussion

In this study, we established and evaluated the NHRI-RP-1 with the aim of optimizing genotype imputation for the Taiwanese population. Increasing the sample size from 1.5K to 2.5K high-coverage genomes significantly improved precision, sensitivity, and F1 score, consistently outperforming TWB1.5K. Notably, in terms of genome-wide consistency, NHRI-RP-1 maintained stable genotype concordance (0.55–0.65) and achieved significantly higher empirical r^2^ values (0.35–0.45) than global reference panels. While performing comparably to TWB1.5K across common variants, NHRI-RP-1 exhibited a distinct advantage for the rare-variants (MAF < 1%), where it attained a concordance rate of approximately 0.3 and an r^2^ of 0.2, nearly doubling the accuracy of TWB1.5K. These findings underscore the value of increasing sample size to capture greater haplotype diversity.

However, the optimal conditions (sample size and sequencing depth) for maximizing performance warrants further studies. For comparison, a Japanese study has integrated the 1KG high-depth panel with 7,472 genomes sequenced at various depths (30 ×, 15 ×, or 3 ×), creating the so-called 1KG +  7 K panel. Their findings demonstrated that this large, hybrid-depth panel achieved imputation accuracy of r^2^ > 0.8 even for variants with a MAF as low as 1% [10]. This comparison highlights two important points: first, the benefit of expanding sample size beyond a few thousand individuals, and second, the potential trade-off between sequencing depth and sample number. While high-coverage sequencing provides more accurate variant calls, increasing the number of genomes—even at moderate or low depth—can substantially enhance imputation accuracy for low-frequency variants. Taken together, these findings suggest that both depth and scale are critical parameters, and future work should aim to define the optimal balance between sample size and sequencing depth that maximizes imputation accuracy in population-specific reference panels.

The allele frequency significantly influenced imputation quality. Stringent thresholds (e.g., MAF ≥ 2 × 10^–4^) enhanced sensitivity and F1 scores across chromosomes, underscoring the importance of rare variant representation in population-specific panels. Rare variants are often geographically and ancestrally unique, and a reference panel that closely matches with the ethnic background of the study cohort ensures higher imputation accuracy and a greater statistical power for genetic association studies. By enabling the accurate imputation of rare, population-specific variants, NHRI-RP-1 supports efforts to uncover “missing heritability” and is able to identify novel genetic associations, thereby advancing biomarker discovery, the development of precise diagnostics, and the creation of targeted therapeutic strategies. In this context, our reference panel and the NHRI Imputation Server align well with the recent efforts of and the results from Taiwan Precision Medicine Initiative (TPMI), where a large number of SNP genotypes have been collected to help define disease and the risks of various traits [24,25]. Integration of these resources should unleash the power of this non-Caucasian genomic data set for the implementation of precision health in Taiwan.

Clinical applications further validated NHRI-RP-1’s utility. The panel achieved near-perfect accuracy when identifying pathogenic variants in genes such as *APOB*, *LDLR*, *NOTCH3*, and *GJB2* and outperformed 1KG Phase 3 in imputing *DRD1* variants, confirming its reliability for both rare disease-related variants and population-specific loci. In GWAS analyses, NHRI-RP-1 produced fewer but potentially more accurate associations compared to 1KG Phase 3 reference panel, suggesting that population-specific reference panels may reduce false positives while focusing on variants of greater relevance to the target population.

## Conclusions

We have developed the population-specific NHRI-RP-1 using 2,561 WGS samples and optimized it for imputation. Larger sample sizes (2.5K) and stringent MAF thresholds (≥ 2 × 10^–4^) improved precision, sensitivity, and the detection of rare variants. Compared with global panels, NHRI-RP-1 achieved superior performance for Taiwanese-specific variants and demonstrated high clinical accuracy for pathogenic variants. These findings establish NHRI-RP-1 as a robust tool for genetic medicine research and for implementing precision medicine, offering both improved discovery power and translational utility within the Taiwanese population. Furthermore, our reference panel has potential values when carrying out similar genomic research targeting at the broad Han Chinese and other related Asian populations, as well as the Chinese diaspora throughout the world.

## Supplementary Information

Additional file 1. A comparative analysis of precision and sensitivity across diverse sample sizes with NHRI-RP-1 dataset. Compare the a. precision and b. sensitivity across chromosomes includes different sample sizes.

Additional file 2. A comparative analysis of precision and sensitivity across minor allele frequencies with the 2.5K NHRI-RP-1 dataset. Compare the a. precision and b. sensitivity across chromosomes with the 2.5K NHRI-RP-1 dataset, which encompasses diverse minor allele frequencies.

Additional file 3. The comparison of a. precision and b. sensitivity with the 2.5K NHRI-RP-1 dataset, characterized by a minor allele frequency 2×10^-4^, is conducted across varying r-squared thresholds. The imputed variants below the r-squared values were excluded.

Additional file 4. A comparative analysis of precision and sensitivity, utilizing 2.5K NHRI-RP-1 (with a MAF ≥ 2×10^-4^, r^2^ ≥ 0), TOPMed, 1KG 30X, 1KG phase 3, and TWB1.5K reference panels. The comparison of a. precision and b. sensitivity with 2.5K NHRI-RP-1, TOPMed, 1KG 30X, 1KG phase 3, and TWB1.5K reference panels by chromosomes.

Additional file 5. A comparative analysis of precision and sensitivity, utilizing 2.5K NHRI-RP-1, TOPMed, 1KG 30X, 1KG phase 3, and TWB 1.5K reference panels. The comparison of a. precision and b. sensitivity with 2.5K NHRI-RP-1, TOPMed, 1KG 30X, 1KG phase 3, and TWB 1.5K reference panels by MAF intervals.

## Data Availability

NHRI-RP-1 is accessible to qualified researchers through an application process via our website (http://imgm.healthhub.tw/NHRI-RP-1.html), and it can be used for genotype imputation. The data supporting this study's findings, processed data, and codes for all analyses are available from the corresponding authors. The TOPMed, Michigan imputation server and TWB data are available through applicaton [6,17].

## Abbreviations

GWAS: Genome-wide association study
NHRI: National Health Research Institutes
NHRI-RP-1: National Health Research Institutes reference panel -1
MAF: Minor allele frequency
WGS: Whole-genome sequencing
SNP: Single-nucleotide polymorphism
LD: Linkage disequilibrium
1KG: 1000 Genomes
TOPMed: Trans-Omics for Precision Medicine
NARD: Northeast Asian Reference Database
TWB: Taiwan Biobank
HALST: Healthy Aging Longitudinal Study in Taiwan
VQSR: Variant quality score recalibration
gVCF: Genomic variant call format
CVDFACTS: Cardiovascular Disease Risk Factors Community Study in Taiwan
TP: True positive
FP: False positive
FN: False negative
P/LP: Pathogenic and likely pathogenic
QC: Quality control
