# Supplementary material for: A population-specific genomic reference panel for Taiwan: NHRI-RP-1
Source: J Biomed Sci. 2026 Aug 11;33:80. doi: 10.1186/s12929-026-01261-y (PMC13459345; doi:10.1186/s12929-026-01261-y)
Supplement: Supplementary file 1 [file 12929_2026_1261_MOESM1_ESM.docx]

**Additional file 1.** A comparative analysis of precision and sensitivity across diverse sample sizes, utilizing the NHRI-RP-1 as a reference panel. Compare the **a.** precision and **b.** sensitivity across chromosomes with the NHRI-RP-1 dataset, which includes diverse sample sizes (0.5K, 1K, 1.5K, 2K, and 2.5K).

**b**

**a**
