# Supplementary material for: A population-specific genomic reference panel for Taiwan: NHRI-RP-1
Source: J Biomed Sci. 2026 Aug 11;33:80. doi: 10.1186/s12929-026-01261-y (PMC13459345; doi:10.1186/s12929-026-01261-y)
Supplement: Supplementary file 2 [file 12929_2026_1261_MOESM2_ESM.docx]

**Additional file 2.** A comparative analysis of precision and sensitivity across minor allele frequency thresholds, employing the 2.5K NHRI-RP-1. Compare the **a.** precision and **b.** sensitivity across chromosomes with the 2.5K NHRI-RP-1 dataset, which exclude variants with minor allele frequencies below thresholds (0.05, 0.01, 0.001, and 2×10^-4^).

**b**

**a**
