# Supplementary material for: A population-specific genomic reference panel for Taiwan: NHRI-RP-1
Source: J Biomed Sci. 2026 Aug 11;33:80. doi: 10.1186/s12929-026-01261-y (PMC13459345; doi:10.1186/s12929-026-01261-y)
Supplement: Supplementary file 3 [file 12929_2026_1261_MOESM3_ESM.docx]

**b**

**a**

**Additional file 3.** The comparison of **a.** precision and **b.** sensitivity with the 2.5K NHRI-RP-1 dataset, excluded variants below a minor allele frequency of 2×10^-4^, is conducted across varying r-squared thresholds (0, 0.3, and 0.5). The imputed variants below the r-squared values (0, 0.3, and 0.5) were excluded.
