# Supplementary material for: A population-specific genomic reference panel for Taiwan: NHRI-RP-1
Source: J Biomed Sci. 2026 Aug 11;33:80. doi: 10.1186/s12929-026-01261-y (PMC13459345; doi:10.1186/s12929-026-01261-y)
Supplement: Supplementary file 5 [file 12929_2026_1261_MOESM5_ESM.docx]

**Additional file 5.** A comparative analysis of precision and sensitivity, utilizing NHRI-RP-1 (2.5K, MAF $\geq$ 2×10^-4^, r^2^ $\geq$ 0), TOPMed, 1KG 30X, 1KG phase 3, and TWB1.5K reference panels. The comparison of **a.** precision and **b.** sensitivity by MAF intervals.

**a**

**b**
